# Mining Fiskeby III and Mandarin (Ottawa) Expression Profiles to Understand Iron Stress Tolerant Responses in Soybean

**DOI:** 10.3390/ijms222011032

**Published:** 2021-10-13

**Authors:** Jamie A. O’Rourke, Michael J. Morrisey, Ryan Merry, Mary Jane Espina, Aaron J. Lorenz, Robert M. Stupar, Michelle A. Graham

**Affiliations:** 1 USDA-ARS, CICGRU, Ames, IA 50011, USA; Michael.Morrisey@usda.gov (M.J.M.); Michelle.Graham@usda.gov (M.A.G.); 2 Department of Genetics and Agronomy, University of Minnesota, St. Paul, MN 55108, USA; merry049@umn.edu (R.M.); espin164@umn.edu (M.J.E.); lore0149@umn.edu (A.J.L.); stup0004@umn.edu (R.M.S.)

**Keywords:** soybean, iron deficiency, RNA-seq, virus induced gene silencing (VIGS)

## Abstract

The soybean (*Glycine max* L. merr) genotype Fiskeby III is highly resistant to a multitude of abiotic stresses, including iron deficiency, incurring only mild yield loss during stress conditions. Conversely, Mandarin (Ottawa) is highly susceptible to disease and suffers severe phenotypic damage and yield loss when exposed to abiotic stresses such as iron deficiency, a major challenge to soybean production in the northern Midwestern United States. Using RNA-seq, we characterize the transcriptional response to iron deficiency in both Fiskeby III and Mandarin (Ottawa) to better understand abiotic stress tolerance. Previous work by our group identified a quantitative trait locus (QTL) on chromosome 5 associated with Fiskeby III iron efficiency, indicating Fiskeby III utilizes iron deficiency stress mechanisms not previously characterized in soybean. We targeted 10 of the potential candidate genes in the Williams 82 genome sequence associated with the QTL using virus-induced gene silencing. Coupling virus-induced gene silencing with RNA-seq, we identified a single high priority candidate gene with a significant impact on iron deficiency response pathways. Characterization of the Fiskeby III responses to iron stress and the genes underlying the chromosome 5 QTL provides novel targets for improved abiotic stress tolerance in soybean.

## 1. Introduction

Iron deficiency chlorosis (IDC) is a major issue in most non-graminaceous crop species around the world. Though iron (Fe) is prevalent in all soils, a variety of factors, including soil composition, moisture, and pH levels, can easily render Fe^2+^ biologically unavailable. Two different strategies have been identified for iron uptake in plant species [1]. Dicot species, including soybean, utilize the strategy I system where protons are secreted into the rhizosphere by ARABIDOPSIS H^+^ ATPase 2 (AHA2) [2] to acidify the soil. This releases iron from various cofactors, and it is reduced into the biologically available Fe^2+^ by FERRIC REDUCTASE OXIDASE 2 (FRO2) [3], which is then transported into the plant root by IRON REGULATED TRANSPORTER 1 (IRT1) [4]. Additionally, strategy I plants actively secrete a number of compounds from roots, including a variety of coumarins [5]. These coumarins may improve iron acquisition by chelating Fe^3+^ and/or reducing Fe^3+^ to Fe^2+^ for transport into plant roots [5]. For a more thorough examination of Strategy I, we recommend the following review articles [6,7,8,9]. Though the quality of seeds and fruit from iron-deficient plants remains unaffected, the quantity is dramatically reduced. In soybean, the second most prevalent crop species grown in the US, even a slight reduction in available iron reduces end of the season yield by 20% [10,11].

The process of identifying genes underlying soybean iron deficiency traits has been slow, largely due to limited genomic tools for functional analysis. Limitations include ease of use, cultivar specificity, and cost. Further, findings from Arabidopsis, the model species in which most iron deficiency studies have been performed, have not directly translated into soybean, likely due to the complex nature of the soybean genome [12]. This is compounded by the selection constraints imposed by breeding to improve soybean yield and quality; constraints that were not experienced by Arabidopsis. In soybean, Lin, et al. [13] identified a major quantitative trait locus (QTL) on chromosome Gm03 responsible for 70% of the phenotypic variation for iron deficiency tolerance. This QTL was identified in every subsequent soybean:iron study, though investigation of the underlying genes has not proven particularly fruitful in improving IDC tolerance. A recent study by our group found this QTL was composed of four distinct regions, each with candidate gene(s) associated with specific aspects of the soybean iron deficiency response; iron uptake, DNA replication and methylation, and defense [14]. While the Gm03 QTL region does not show genetic variation in modern elite lines [15], the 2020 genome wide association study (GWAS) also showed the soybean germplasm collection likely contains multiple iron deficiency mechanisms. This finding was re-affirmed by Merry et al. [15], finding resistance to iron deficiency stress was associated with a QTL on Gm05, which is genetically variable within elite cultivars [15]. The QTL on Gm05 [15] overlaps with two regions identified in the Assefa et al. [14] IDC GWAS study (*Glyma.05G000100*-*Glyma.05G001300* and *Glyma.05G001700-Glyma.05G002300*). Because the region on Gm05 is not fixed in elite breeding material, it holds promise for improving IDC tolerance. Identifying a candidate gene conferring iron deficiency stress tolerance would be ideal, as that gene could be utilized in either traditional breeding or transgenic approaches for soybean improvement. Accordingly, Merry et al. [15] fine mapped the Gm05 IDC QTL to a 137 kb region containing 17 protein coding sequences and identified the two most promising candidate genes underlying this QTL region: *Glyma.05G001400*, encoding a VQ-domain containing protein, and *Glyma.05G001700*, which encodes a MATE transporter.

Virus-induced gene silencing (VIGS) is a simple method to knock down gene expression of targeted candidate genes [16]. This reverse genetic tool has been used to validate candidate genes underlying numerous traits, including resistance to Asian soybean rust [17,18], iron deficiency chlorosis [19], drought [20], and soybean cyst nematode resistance [21]. Utilizing VIGS to characterize candidate genes is a relatively quick and inexpensive method to screen a relatively large number of candidate genes to determine if down-regulation of candidate genes results in a visible phenotypic change. Previous publications have illustrated the utility of coupling VIGS with whole-genome expression analyses to understand the changes in gene expression and molecular networks associated with the silenced gene [17,19,22,23].

The object of this study was to utilize RNA-seq to investigate the gene expression differences in Fiskeby III (iron deficiency tolerant) and Mandarin (Ottawa, iron deficiency susceptible) grown in iron sufficient (FeS, 100 µM Fe(NO_3_)_3_) and iron-deficient (FeD, 50 µM Fe(NO_3_)_3_) hydroponic conditions. This was coupled with phenotypic analyses of VIGS plants followed by RNA-seq analysis of Fiskeby III VIGS silenced plants to determine how silencing of the candidate gene, *Glyma.05G001700*, altered the Fiskeby III iron deficiency stress response. This powerful approach allows us to identify molecular networks associated with Fiskeby III iron deficiency tolerance and the potential role for *Glyma.05G001700* in that process

## 2. Results

### 2.1. Phenotypic Analyses

**Figure 1:**
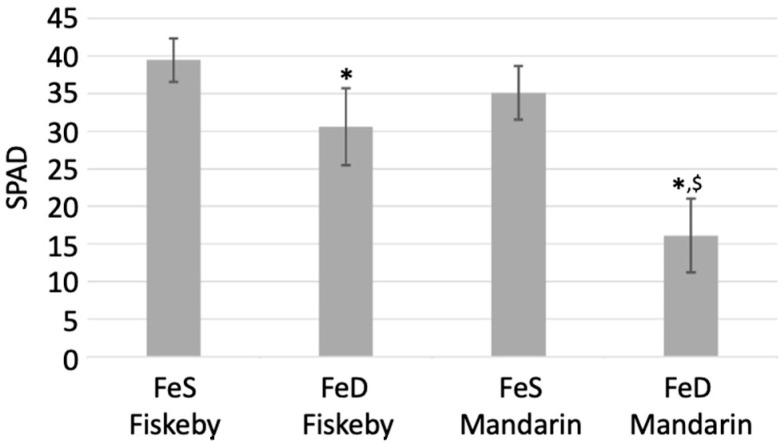
SPAD readings comparing Fiskeby III and Mandarin (Ottawa) grown for 14 days in iron sufficient (FeS) and iron-deficient (FeD) hydroponic conditions. Error bars are standard deviations calculated from six biological replicates. Astrisk (*) indicates a statistical difference between FeS and FeD within the same genotype. ^$^ indicates a difference between genotypes of the same iron treatment (between Fiskeby III and Mandarin in FeD).

Fiskeby III is reported to be more tolerant to a variety of abiotic stresses than most soybean germplasm [24,25,26,27,28]. Two studies have shown Fiskeby III to be tolerant to, though not completely resistant, to FeD stress. After 16 days of FeS (100 µM Fe(NO_3_)_3_) or FeD (50 µM Fe(NO_3_)_3_) hydroponic conditions, Fiskeby III and Mandarin (Ottawa) showed very different phenotypic responses (Figure 1). There were no statistical difference in soil-plant analysis development (SPAD) chlorophyll readings between Fiskeby III and Mandarin (Ottawa) in FeS conditions. However, under FeD conditions, Fiskeby III SPAD readings dropped 8.9 points, which was statistically lower than FeS Fiskeby III, but not different from FeS Mandarin (Ottawa). As expected, under FeD conditions Mandarin (Ottawa) exhibited severe chlorosis, with SPAD measurements dropping 19 points, statistically different from both Fiskeby III and Mandarin (Ottawa) in FeS and from Fiskeby III in FeD conditions (Figure 1).

**Figure 2:**
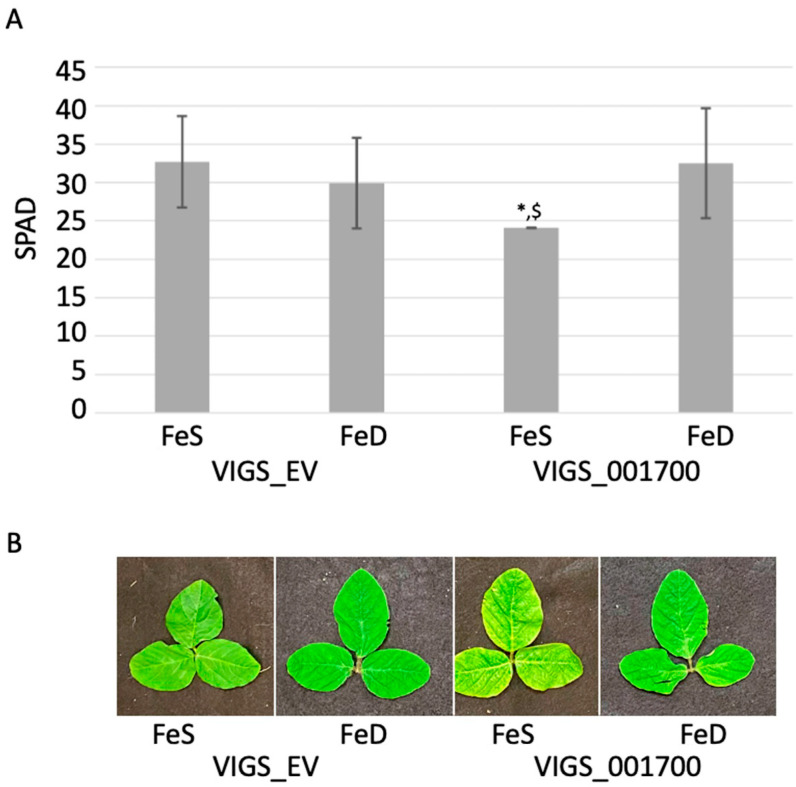
Phenotypic analysis of Fiskeby III infected with either empty vector or *Glyma.05G001700* VIGS constructs in FeS and FeD hydroponic conditions. (**A**). SPAD readings at 14 days in hydroponics. Error bars are standard deviations calculated from six biological replicates. * indicates statistically significant differences between VIGS_Glyma.05G001700 in FeS and FeD conditions; ^$^ indicates statistically significant differences between VIGS_Glyma.05G001700 in FeS and VIGS_EV in FeS. (**B**). Photographs of representative V4 trifoliates.

As previously indicated, the Gm05 IDC QTL corresponds to 17 protein-encoding genes [15]. Of these, 13 were expressed in shoots and roots based on published RNA-seq atlases [29,30]. Based on expression, gene duplication, annotations, and genic structure, 10 genes were considered good targets for VIGS analysis. Following testing in both soil and hydroponic (FeS and FeD) conditions, only a single VIGS construct, corresponding to *Glyma.05G001700*, exhibited phenotypes consistent with altered iron stress tolerance. These included increased interveinal chlorosis under FeS conditions, which corresponds to decreased SPAD readings, but no statistically significant change in SPAD readings under FeD conditions compared to controls (Figure 2).

### 2.2. SNP Analysis of Genotypes of Interest

**Figure 3:**
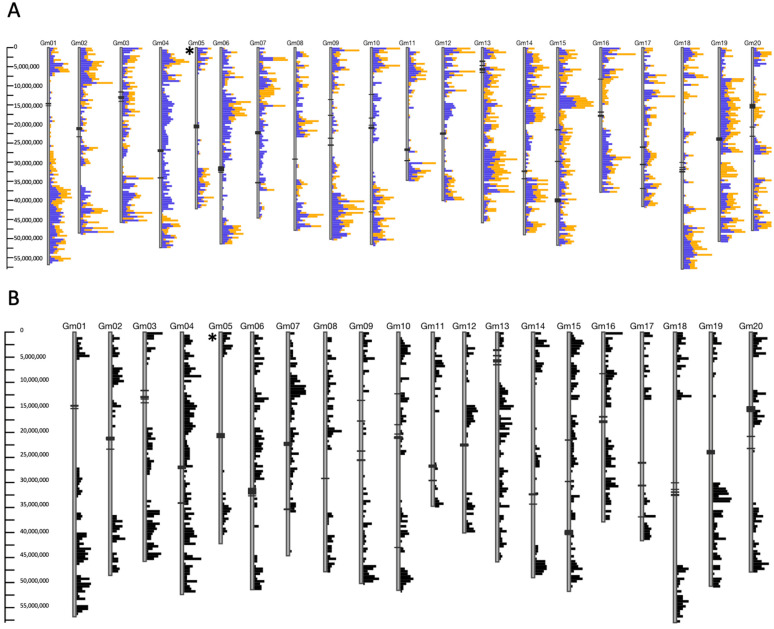
SNP profiles of Clark, Fiskeby III, and Mandarin (Ottawa). Each of the 20 chromosomes in soybean (*Glycine max*) is represented by a vertical line. SNP differences are represented by horizontal lines. Only SNPs that differ between the reference genotype and samples of interest are shown. The more SNP differences within a chromosomal region, the longer the line. The asterisk to the left of Gm05 indicates the location of the Gm05 iron efficiency QTL of interest [15]. (**A**). SNP differences between Clark and Fiskeby III (blue) and between Clark and Mandarin (Ottawa) (orange). (**B**). SNP differences between Fiskeby III and Mandarin (Ottawa).

Soybean has a notoriously narrow genetic base due to a historical genetic bottleneck [12,31]. The genotypes Mandarin (Ottawa) and Fiskeby III are both optimized for northern growing regions and are both plant introduction (PI) lines, originally collected from China and Sweden, respectively [32]. Given our knowledge base of how iron deficiency alters gene expression in the iron stress-tolerant genotype Clark, we were interested in how similar either line was to Clark. Using the Genotype Comparison Tool (GCViT) available at SoyBase (https://www.soybase.org/gcvit/, accessed on 19 May 2021), we confirmed Fiskeby III and Mandarin (Ottawa) were more similar to each other than they were to Clark (Figure 3A). However, the SNP patterns on Gm05 reflect the contrasting IDC phenotypes and identification of the Gm05 IDC QTL from a Fiskeby III × Mandarin (Ottawa) biparental population (Figure 3B). The diversity reflected in the SNP analyses supports the hypothesis proposed by Assefa et al. [14]; that multiple mechanisms conferring tolerance to iron deficiency stress were present in the soybean germplasm collection. Given the novel QTL identified in Fiskeby III and the genotypic differences of Fiskeby III compared to Clark, it is highly possible that Fiskeby III utilizes different iron sensing, uptake, or homeostatic mechanisms than Clark.

### 2.3. RNA-Seq Analysis

**Figure 4:**
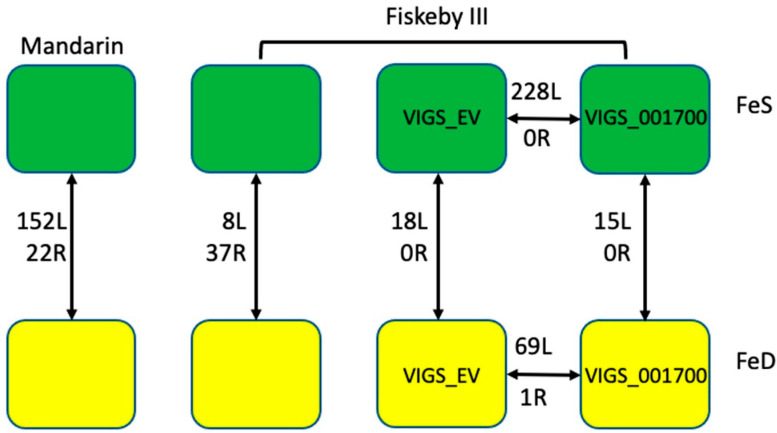
Experimental Design. Green represents iron sufficient (FeS, 100 μM Fe(NO_3_)_3_). Yellow represents iron deficiency (FeD, 50 μM Fe(NO_3_)_3_). Only one set of Mandarin (Ottawa) plants was included in this experiment. These were not inoculated with any VIGS construct. Plants inoculated with VIGS_Glyma.05G001700 are denoted as VIGS_001700. Results from edgeR DEG analyses (required to have FDR < 0.01) are indicated by numbers followed by either an L (leaf) or R (root), to indicate the tissue analyzed.

Seeds of both Fiskeby III and Mandarin (Ottawa) were surface sterilized and placed on germination paper with deionized water. After 7 days, plants were transferred to either FeS or FeD hydroponic conditions, where they were maintained for 16 days until plants reached the V4 stage. Two days after transfer to hydroponics, ¼ of Fiskeby III in both FeS and FeD hydroponics were rub inoculated with VIGS_Glyma.05G001700 construct and ¼ of Fiskeby III were rub inoculated with VIGS_EmptyVector (VIGS_EV) construct. SPAD readings and height measurements were taken 14 days after VIGS inoculation. After phenotyping, V4 trifoliate and root systems were collected and flash-frozen in liquid nitrogen for RNA extraction. This approach allowed us to grow all plants used in this experiment simultaneously. RNA sequencing was conducted at the Iowa State University DNA facility. Using the analyses pipelines described in the materials and methods, we identified differentially expressed genes (DEGs) responding to iron stress in Mandarin (Ottawa), and Fiskeby III leaves and roots (Figure 4). The same bioinformatic approach was used to compare gene expression profiles in leaves and roots of VIGS_EV and VIGS_Glyma.05G001700 plants to determine the impact of silencing *Glyma.05G001700* on gene expression profiles in both iron sufficient and deficient growth conditions (Figure 4).

#### 2.3.1. *Mandarin* RNA-Seq

**Figure 5:**
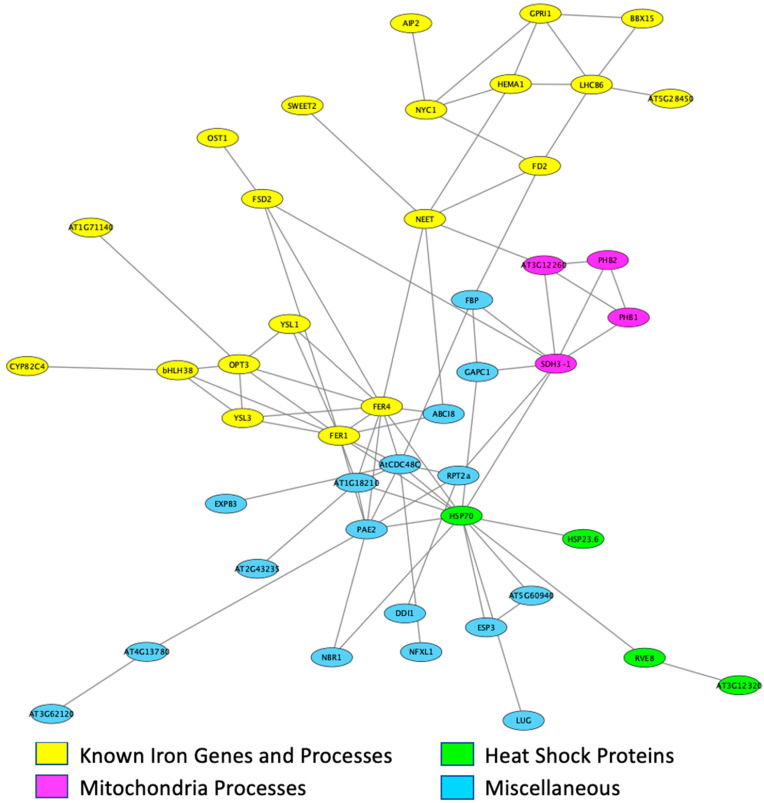
STRING network for DEGs identified between FeS and FeD in Mandarin (Ottawa) leaves. The 152 DEGs identified in Mandarin (Ottawa) responding to iron deficiency correspond to 122 genes in *Arabidopsis thaliana*. Known interactions between the Arabidopsis homologs were identified using the STRING database. Only 44 of the 122 Arabidopsis genes were known to interact in any way. Annotations of Arabidopsis genes were used to assign biological functions to the genes, these are denoted in the legend.

We identified 152 DEGS in iron stress susceptible Mandarin (Ottawa) leaves responding to iron stress (Figure 4, Table S1), including 21 transcription factors (TFs). Gene ontology (GO) analyses identified three significantly (Corrected *p*-value ≤ 0.05) over-represented GO terms; iron ion homeostasis (GO:0055072), response to iron ion (GO:0010039), and cellular iron ion homeostasis (GO:0006879). To gain further insight into the function of these 152 DEGs, we took advantage of STRING (string-db.org) [33,34] to analyze the 122 corresponding Arabidopsis best homologs. Of these, 44 formed a single network (protein–protein interaction (PPI) *p*-value = 3.26 × 10^−6^) of known interactions (Figure 5). The network was centered on multiple ferritin proteins and other proteins known to be involved in iron uptake and homeostasis (including bHLH038 *At3g56970*), YSL (*At4g24120* and *At5g53550*), OPT3 (*At4g16370*), NEET (*At5g51720*), GPRI1 (*At2g20570*), and BBX15 (*At1g25440*) by directly or indirectly interacting with the ferritin genes. Although only represented once in the STRING analysis, both homologs of AtbHLH038 (*At3g56970*, *Glyma.03G130400*, and *Glyma.03G130600*), located within the canonical IDC QTL on Gm03, were up-regulated due to iron deficiency stress. In Arabidopsis, NEET (*At5g51720*) is an important Fe assimilation protein, known to be directly regulated by AtPYE (*At3g47640*) and AtbHLH104 (*At4g14410*), both important players in the Arabidopsis iron homeostasis network [35,36,37,38]. In Mandarin(Ottawa), NEET homologs (*Glyma.09G091002, Glyma13G193600*, and *Glyma15G231900*) were all down-regulated by iron-deficient conditions. In addition, notable, though not represented in GO or String-db analyses, were seven NAC TFs, one-third of all DE TFs, all of which were up-regulated by FeD stress.

Only 22 iron stress responsive DEGs, and only a single TF (*Glyma.02G008200*), were identified in the roots of Mandarin (Ottawa) plants (Figure 4, Table S2). Annotations associated with these genes were largely uninformative (six had no known annotations), and given the small sample size, neither GO or STRING analyses were appropriate. However, annotations identified three vacuolar iron transporter (VIT) genes (*Glyma.08G076100, Glyma.05G121300*, and *Glyma.08G075900*), all three of which were up-regulated under iron-deficient conditions. Work in other species has shown VIT proteins play an important role in Fe homeostasis and that upregulation of different VIT proteins can improve Fe accumulation under FeD conditions [39,40]. Down-regulated under iron-deficient conditions was *Glyma.15G251300*, which was homologous to AtNAS1 (*At5g04950*). Nicotianamine produced by NAS1 forms complexes with Fe, which play a central role in long-distance Fe transport; usually from shoots to roots, but more recently shown from root to shoots, thus improving growth under FeD conditions [41]. In both soybean and sweet potato, over-expression of NAS1 promotes iron accumulation and IDC tolerance in calcareous soils [42,43].

#### 2.3.2. Fiskeby RNA-Seq

In contrast to Mandarin, only eight genes (seven up-regulated and one down-regulated in FeD) were differentially expressed in iron stress-tolerant Fiskeby III leaves in response to FeD stress (Figure 4 and Figure S1A, Table S3). This suggests that Fiskeby III has largely acclimated to iron stress conditions in the leaves. Two of the eight genes had no obvious annotation leaving only six genes to investigate. One of the six DEGs, the only TF, was *Glyma.03G130400*, a homolog of AtbHLH038 (*At3g56970*), upregulated under FeD conditions. The remaining genes include *Glyma.04G179500*, a homolog of AtGASA14 (*At5g14920*), which regulates abiotic stress resistance by modulating reactive oxygen species accumulation in leaves [44]. The conserved expression pattern in both soybean and Arabidopsis, up-regulated under FeD conditions, suggests it is performing a similar function in Fiskeby III. In addition, up-regulated due to iron deficiency in Fiskeby III leaves is a homolog of AtNAS2 (*At5g56080*). Similar to AtNAS1, AtNAS2 is also involved in nicotianamine biosynthesis and functions in the long-distant transport of iron, zinc, and other metals [45]. The other DEGs include a cellulose synthase (the only down-regulated gene), ATPase, and a major facilitator superfamily protein. Members of the major facilitator superfamily are transporters involved in peptide and hormone transport. Recent studies have also involved members in mediating resistance to various stresses [46,47,48,49].

We identified 37 iron stress-responsive DEGs in roots of Fiskeby III, including four TFs (Figure 4 and Figure S1B, Table S4). GO analyses identified two significantly (corrected *p*-value ≤ 0.05) over-represented GO terms; GO:0042754, negative regulation of circadian rhythm (2 genes) and GO:0043433, negative regulation of sequence-specific DNA binding transcription factor activity (2 genes). The same two genes were assigned to both terms; *Glyma.03G261800* and *Glyma.19G260900*. Both are MYB transcription factors homologous to the Arabidopsis *LHY1* gene, which is involved in the circadian clock. Previous studies have suggested the circadian clock functions as a hub to balance energy requirements for growth and stress tolerance [50,51]. Specifically, FeD stress in soybean effectively pauses the circadian clock to extend iron uptake periods [50]. Given the lack of insights from over-represented GO terms, we examined the annotations of the remaining 35 genes. Obviously associated with FeD stress is *Glyma.12g237367*, a homolog of AtFRD3 (*At3g08040*), and *Glyma.08g076100*, which encodes a vacuolar iron transport (VIT) protein. In Arabidopsis, FRD3 transports citrate into the xylem, which chelates the iron as it is transported to leaf tissues [52]. VIT proteins sequester excess iron into the vacuole. Under FeD conditions, transcripts encoding VIT1 were down-regulated. In addition, down-regulated were transcripts encoding NAS1 (*Glyma.15g251300*). Other non-canonical genes involved in both iron homeostasis and stress tolerance were also differentially expressed in Fiskeby III roots in responses to FeD stress. These include up-regulation in FeD of *Glyma.13g168700*, which encodes a formate dehydrogenase gene known to be responsible for regulating Fe homeostasis and which might mediate stress responses [53]. In addition, up-regulated under FeD was *Glyma.08g169100*, which is involved in fraxetin biosynthesis. Fraxetin, a specific type of coumarin, extends the pH range for efficient Fe^3+^ reduction, improving iron availability in calcareous soils [54]. The remaining DEGs were associated with either stress tolerance, photosynthesis, or growth and development. Identification of a number of canonical iron stress genes suggests Fiskeby III is still actively monitoring iron stress conditions.

### 2.4. VIGS Plants

#### 2.4.1. Phenotypic Analysis of VIGS Plants

In VIGS silenced plants, there is more phenotypic variation, even within plants infected with the same silencing construct. Infection efficacy for all 10 candidate gene VIGS constructs were tested in Williams82 and Clark. Both genotypes exhibited good infection, but no statistically significant phenotypic changes were observed in either soil or hydroponics (data not shown). Preliminary experiments determined Fiskeby III was susceptible to VIGS infection. Accordingly, Fiskeby III was infected with the VIGS_EV construct, and VIGS constructs corresponding to the 10 transcriptionally active genes within the Gm05 QTL. Of all 10 VIGS constructs, the soil-grown plants infected with VIGS_Glyma.05G001700 construct had lower SPAD readings than VIGS_EV infected plants at the third trifoliate (data not shown). Repeating the experiment in FeS and FeD hydroponics found that at 14 days post-FeD stress SPAD readings of VIGS_EV plants grown in FeS and FeD were nearly identical, reinforcing the iron deficiency tolerance of this genotype as demonstrated in previous experiments. Again, the phenotype of VIGS_Glyma.05G001700 infected plants in FeS mirrored the phenotype of soil-grown plants, with statistically lower SPAD readings compared to FSe VIGS_EV. However, for FeD VIGS_Glyma.05G001700 silenced plants SPAD readings were comparable to VIGS_EV plants and statistically higher than FeS VIGS_Glyma.05G001700 grown plants (Figure 2A,B).

#### 2.4.2. Identifying DEGs between VIGS_EV and VIGS_Glyma.05G001700

To understand genes affected by *Glyma.05G001700* silencing in Fiskeby III, we compared VIGS_EV to VIGS_Glyma.05G001700 in FeS and FeD conditions. Because all plants were infected with the bean pod mottle virus (BPMV), these comparisons were similar to comparing near-isogenic lines since the only difference was the silencing of *Glyma.05G001700*. However, this comparison will allow us to identify downstream genes whose expression is directly or indirectly impacted by *Glyma.05G001700* silencing. Importantly, under FeS conditions, this comparison provides a global view of the role Glyma.05G001700 plays in the plant, not just the role of *Glyma.05G001700* in Fe homeostasis. These analyses identified 228 DEGs in FeS leaves and 69 DEGs in FeD leaves (Figure 4 and Figure S1C, Tables S5 and S6). Remarkably, four DEGs were identified in both FeS and FeD conditions; a glutathione S-transferase (*Glyma.10G19290*), a pathogenesis-related protein (AtPBR1, *Glyma.15G062500*), an atypical bHLH TF (*Glyma.01G108700*), whose homolog AtPAR1 (*At3g54040*) is involved in the shade avoidance system [55] and *Glyma.06G306900*, with no known function or Arabidopsis homolog. All four genes were up-regulated in VIGS_Glyma.05G001700 silenced plants in both FeS and FeD conditions when compared to VIGS_EV. There were no DEGs identified in roots of FeS plants, and only a single DEG in FeD roots (*Glyma.01G175200*), a sulfite exporter. This could suggest that *Glyma.05G001700*’s role is iron acquisition and homeostasis is largely restricted to leaves. However, an alternative hypothesis is that leaves are responding to lack of iron because *Glyma.05G001700* is unable to fulfill its role in the roots.

Analyses of the 228 DEGs identified in leaves between VIGS_EV and VIGS_Glyma.05G001700 grown in FeS conditions (Figure 4) identified nine significantly over-represented gene ontology (GO) terms (Table 1). Despite plants being grown in FeS conditions, two of the GO terms were associated with iron homeostasis (GO:0055072 and GO:0006879, 6 genes total), and four were associated with phosphate starvation and homeostasis (GO:0016036, GO:0030643, GO:0019375, GO:0006817, 17 genes total). The remaining three GO terms were associated with photosynthesis (GO:0015979, 13 genes), response to zinc ion (GO:0010043, 7 genes), and generation of precursor metabolites and energy (GO:0006091, 7 genes). While it is important to remember that *Glyma.05G001700* may play a role in molecular networks not associated with Fe, the identification of two over-represented GO terms associated with Fe is notable and provides further evidence that *Glyma.05G001700* is the candidate gene underlying the Gm05 QTL. Among the 6 genes associated with iron homeostasis is a homolog of AtBRUTUS (BTS, *Glyma.09G115100*), which was down-regulated in VIGS_Glyma.05G001700 compared to VIGS_EV. Other iron homeostasis genes include four genes that encode ferritin proteins (FER1, *Glyma.01G124500*, *Glyma.03G050100*, *Glyma.07G155200*, and *Glyma.18G205800*) and two heavy metal transport genes (*Glyma.07G065800* and *Glyma.16G032400*), all of which are up-regulated in VIGS_Glyma.05G001700 plants compared to VIGS_EV plants. Genes obviously involved in phosphate homeostasis include three homologs of phosphoenolpyruvate carboxylase 1 (PEPC1: *Glyma.08G195100*, *Glyma.08G195000*, and *Glyma.07G011800*), three SPX homologs (*Glyma.17G114700*, *Glyma.01G135500*, *Glyma.03G032400*), four purple acid phosphatase genes (*Glyma.05G138400*, *Glyma.06G028200, Glyma.08G056400*, and *Glyma.05G247900*) and homologs of Phosphate 1 (PHO1, *Glyma.10G004800*) and Phosphate 2 (PHO2, *Glyma.13G239100*). All of these, except PHO2, are up-regulated in VIGS_Glyma.05G001700 compared to VIGS_EV under FeS conditions. Analysis of the 170 unique Arabidopsis gene IDs associated with the 228 DEGs using STRING [33,34] identified a gene network (PPI *p*-value = 1.11 × 10^−16^) of 80 Arabidopsis proteins centered on the two thioredoxin homologs (TRX1 (*At3g51030*) and TRX2 (*At5g39950*), corresponding to *Glyma.17G254200* and *Glyma.18G127400*, respectively). From these two genes, four branches form four unique gene clusters; a photosynthesis cluster, an RNA processing cluster, a stress response cluster, and an iron and phosphorus homeostasis cluster (Figure S2). These clusters likely represent the major processes associated with *Glyma.05G001700* in Fiskeby III under sufficient (FeS) conditions.

Under FeD growth conditions, only 69 DEGs were identified in leaves and only a single DEG in roots between VIGS_EV and VIGS_Glyma.05G001700 (Figure 4, Tables S6 and S7). No GO terms were over-represented among the 69 genes. Examining the annotations of the 69 DEGs identified homologs of IRT3 (*Glyma.06G052000*), bHLH038 (*Glyma.03G130600*), and two homologs of SWEET12 (*Glyma.05G202600* and *Glyma.08G009900*) are all differentially expressed. While homologs of *IRT3* and *SWEET12* were up-regulated in VIGS_Glyma.05G001700, the homolog of *bHLH038* was down-regulated compared to VIGS_EV. IRT3, SWEET12, and bHLH038 are all known to play a role in iron homeostasis under FeD in Arabidopsis and have been identified in Clark iron stress studies in soybean [56,57,58,59]. A STRING analysis of the Arabidopsis homologs finds 23 Arabidopsis genes linked in a network (PPI *p*-value = 6.68 × 10^−9^, Supplemental Figure S3). A smaller, independent, network includes IRT3 (*Glyma.06G052000*), NRAMP3 (*Glyma.11G051500*), ZIP1 (*Glyma.20G063100*), ABCC3, a multi-drug resistance protein (*Glyma.05G145000*, which transports glutathione conjugates into vacuoles [60]) and bHLH38 (*Glyma.03g130600*). IRT3, NRAMP3, and ZIP1 are all up-regulated in VIGS_Glyma.05G001700, while MRP3 and bHLH38 are down-regulated. No DEGs were identified in roots between VIGS_EV and VIGS_Glyma.05G001700.

#### 2.4.3. DEGs VIGS_EV Response to Iron Treatment

As infection with the VIGS vector can result in phenotypic and gene expression changes, it is important to confirm that Fiskeby III plants infected with the BPMV_EV, still respond to iron stress as expected. Comparing VIGS_EV plants in iron sufficient and deficient conditions identified 18 genes that were differentially expressed in leaves at 14D post-VIGS 16 D post iron stress, but no genes were differentially expressed in roots (Figure 4, Table S8). Of the 18 DEGs in VIGS_EV leaves only one gene, *Glyma.03g130400*, one of the two bHLH038 (*At3g56970*) homologs were also differentially expressed in normal Fiskeby III leaves in response to iron treatment. In VIGS_EV, both soybean *bHLH038* homologs are up-regulated in leaves under FeD conditions. The two soybean *bHLH038* homologs lay within the Gm03 IDC QTL and were proposed by Peiffer et al. [61] as the candidate genes underlying the Gm03 QTL. Four additional stress-related genes are also up-regulated under FeD conditions. These include *Glyma.04G228300*, a homolog of AtAPRR5 (*At5g24470*), which is involved in the SnRK pathway and regulates cytokinin [62]; *Glyma.05G145000*, a member of the multidrug resistance-associated protein family likely serving as a metal transporter in Arabidopsis (*At3g13080*) [60]; *Glyma.05G169900*, encodes a plantacyanin a copper-containing protein involved in tolerance to heavy metal stress tolerance in Arabidopsis (At2g02850) [63]; *Glyma.10G276700*, a major facilitator known to transport nitrate in Arabidopsis (*At2g39210*) [64], and *Glyma.20G133200*, a homolog of AtZAT10. In Arabidopsis, ZAT10 (*At1g27730*) is strongly induced by ABA and numerous abiotic stresses to provide enhanced stress tolerance [65]. The Arabidopsis homologs of three additional genes are clearly associated with stress response pathways. *Glyma.06G261100* is homologous to *At4g27290*, a member of the receptor-like kinase family, which is known to play a role in defense [66]. The Arabidopsis homolog of *Glyma.14G223000* (*At1g76160*) regulates the balance between growth and autophagy under stress [67], and the expression of *At5g15230*, which is the homolog of *Glyma.19G022500* is repressed by stress hormones [68]. Given the annotations of their Arabidopsis homologs, it is highly probable all three of these genes are also associated with stress response pathways, but only *Glyma.06G261100* is up-regulated by FeD, the other two are down-regulated. Annotations of the remaining eight DEGs include three unknowns, four involved in cell wall biosynthesis and one associated with the circadian clock. These findings suggest that the VIGS vector had little impact on the Fiskeby III iron stress response. Observed differences between VIGS and non-VIGS plants are likely due to responses to viral infection.

#### 2.4.4. VIGS_Glyma.05G001700 Response to Iron Treatment

If *Glyma.05G001700*, a MATE transporter, is the candidate gene underlying the Gm05 IDC QTL in Fiskeby III, then silencing the gene using VIGS should alter the gene expression of genes involved in Fe response pathways. Comparing RNA-seq profiles of VIGS_Glyma.05G001700 plants grown in FeS and FeD found 15 DEGs in leaves, but no DEGs in roots (Figure 4, Table S9), a tissue expression pattern similar to DEG analysis of VIGS_EV infected plants. However, none of the genes differentially expressed in VIGS_Glyma.05G001700 plants were differentially expressed in VIGS_EV plants (Figure S1D). Further, 5 of the 15 VIGS_Glyma.05G001700 DEGs are known to be associated with phosphate deficiency (-P_i_) responses, not FeD, which is reflected in the three over-represented GO terms (Table 2). Phosphate response genes include a purple acid phosphatase (*Glyma.05g247900*), two pyridoxal phosphate phosphatase-related proteins (*Glyma.08g195000* and *Glyma.08g195100*), a SQDG2 homolog (*Glyma.03g078300*), and an SPX homolog (*Glyma.17g114700*), all of which are down-regulated under FeD conditions. The remaining genes either have no known annotations (4) or are associated with senescence (2), defense (3), or cell wall integrity (1). Failure to identify canonical iron stress response genes in *Glyma.05G001700* silenced Fiskeby III plants suggests silencing prevented the normal iron stress responses we observed in non-silenced Fiskeby III. Further, it suggests Fiskeby III plants unable to induce iron stress responses can induce phosphate stress responses, perhaps explaining Fiskeby III tolerance to multiple abiotic stresses.

## 3. Discussion

### 3.1. Comparing Mandarin (Ottawa) and Fiskeby III Gene Expression

After 16 days of exposure to FeD stress, the initial FeD stress response has already occurred. In Clark, the genotype used for the majority of soybean iron deficiency studies, gene expression changes have been observed as early as 30 min after iron stress is applied [59]. The extended time of stress exposure in our experiment likely explains why none of the DEGs in any of our analyses correspond to the IDC QTL on chromosome Gm05. Instead, the DEGs identified in this manuscript are downstream, perhaps long-term responses to extended FeD stress conditions. Mandarin (Ottawa) has more DEGs in response to FeD than Fiskeby III, suggesting the two genotypes have different FeD response mechanisms. However, two genes in leaves and seven genes in roots are differentially expressed in both Mandarin (Ottawa) and Fiskeby III in response to FeD stress (Figure S1A,B). In leaves, the two genes are *Glyma.03G130400* and *Glyma.13G068200*, and both genes are up-regulated under FeD in both genotypes. *Glyma.03G130400* is one of two homologs of *AtbHLH038* located within the historical IDC QTL on soybean chromosome Gm03. In Arabidopsis, this protein (*At3g56970*, bHLH038) interacts with FIT to regulate iron uptake [57], but VIGS silencing of this gene has not revealed a major role in FeD tolerance in the soybean genotype Clark [14]. Given the genotypic differences between Clark and the two genotypes in this study (Figure 3), it is possible that in the Mandarin (Ottawa) and Fiskeby III genetic backgrounds, the role of bHLH038 in FeD responses more closely resembles that of Arabidopsis. The other gene, *Glyma.13G068200* is a major facilitator superfamily protein and members of this gene family in Arabidopsis are associated with various aspects of FeD tolerance [46,47,48]. The seven genes DE in roots of both Mandarin (Ottawa) and Fiskeby III (*Glyma.01G129200, Glyma.01G130800, Glyma.05G204600, Glyma.08G076100, Glyma.14G032000, Glyma.14G20500*, and *Glyma.15G251300*) all exhibited the opposite expression in Fiskeby III compared to Mandarin (Ottawa). Among these seven genes, the most notable is *Glyma.15G251300*, which encodes NAS1. In Fiskeby III NAS1 expression is down-regulated in FeD grown plants, while in Mandarin (Ottawa), it is up-regulated. This example might demonstrate that Fiskeby III has recognized the nutrient limitation and has achieved a new homeostatic level at 16 D of FeD, while Mandarin (Ottawa) is still attempting to take up Fe from the environment and move it to the leaf tissues for use in photosynthesis. A study by Atencio et al. [69] reported that in iron efficient Clark, the number of DEGs and the magnitude of their expression increased with increasing duration of iron stress (from two to 10 days). In contrast, iron inefficient Isoclark had fewer DEGs, and the direction of expression largely reversed between 2 and 10 days of iron stress. Much like Fiskeby III, Clark did not appear to be responding to the iron stress in the leaves, with only five DEGs identified at 2 days post-iron stress. Similarly, DEGs identified in Clark roots at two days post-iron stress was also associated with iron uptake and homeostasis. However, an important difference between Clark and Fiskeby III is that across multiple timepoints [59,69,70], Clark represses growth by inhibiting pathways associated with DNA replication, cell division, and development. This is likely further evidence that Clark and Fiskeby III utilize different iron stress tolerance mechanisms, likely governed by the two disparate IDC QTLs. Understanding these differences between Clark and Fiskeby III is critically important for protecting yield under iron stress conditions.

### 3.2. Gene Expression in Mandarin (Ottawa) Leaves and Roots

In Mandarin (Ottawa), the 152 DEGs identified in leaves due to iron stress (Figure 4) are associated with three over-represented GO terms, all related to iron homeostasis. However, these GO terms only reflect 12 DEGs. A STRING analysis produces a network that incorporates these 12 DEGs plus an additional 44 genes into a network centered on ferritin encoding genes (Figure 5). Interacting gene clusters related to heatshock proteins, iron stress, and mitochondrial respiration extend from the ferritin center. While GO terms only identified 12 genes associated with iron processes, the STRING network identified 19 genes with functions directly related to iron homeostasis. The precise role and importance of the genes not included in the STRING network in the Mandarin (Ottawa) iron deficiency response is unclear as over 30 of the genes have no known function in Arabidopsis, and the remaining genes are associated with a wide variety of responses, including cell wall structure and transport. Of note are transcription factors not included in the STRING analyses. Only two of the four DE MYB and one of the seven NAC TFs DE in Mandarin (Ottawa) leaves are included in the STRING analysis. The Arabidopsis homologs of all four DE MYB TFs are associated with stress responses; *Glyma.01G217500* (MYB3R5, *At5g02320*) inhibits cell division in response to DNA damage, *Glyma.02G264900* (MYB73, *At4g37260*) is associated with salinity tolerance, *Glyma.10G048500* (REVEILLE8, *At3g09600*) is involved in heat shock responses, and *Glyma.12G117700* (GPRI1, *At2g20570*) affects ozone tolerance and activates JA dependent disease susceptibility and immunity [71,72,73,74,75,76]. The seven soybean NAC genes correspond to four Arabidopsis NAC homologs and are all up-regulated by FeD conditions. *Glyma.14G084300* and *Glyma.17G240700* (NAC011, *At1g32510*) enhance tolerance to drought and cold stress [77]. *Glyma.02G222300, Glyma.07G048000*, and *Glyma.16G016700* (NAC9, *At4g35580*) are associated with osmotic stress signaling and plant immunity [78,79]. *Glyma.07G048100* (NAC1, *At3g49530*) regulates ER stress-responsive genes, and *Glyma.19G002900* (NAC44, *At3g01600*) links various stress responses and signaling pathways [80,81,82]. Previous work by our group [83] has demonstrated the importance of NAC TFs in the Clark genotype FeD response. The DE of seven NAC TFs in Mandarin (Ottawa) leaves indicates the NAC TF family also plays an important role in the Mandarin (Ottawa) FeD genotypic response. It is possible the DE NAC TFs may indicate conserved iron (or abiotic stress) responses within the soybean germplasm. In the roots, 22 genes are DE in response to FeD stress. In general, genes involved in internal iron transport (VIT proteins and NAS1) are up-regulated by FeD. In addition, up-regulated is an acid phosphatase (At2g38600, *Glyma.16G220700*) normally associated with -P_i_ stress responses. Conversely, genes that might play a role in heavy metal uptake (*Glyma.16G178500, Glyma.19G255500*) or abiotic stress responses (*Glyma.15G015100*) are down-regulated by FeD [84,85]. These expression patterns further demonstrate that Mandarin (Ottawa) is actively trying to initiate iron stress responses, but phenotypic differences between Fiskeby III and Mandarin (Ottawa) suggest Mandarin (Ottawa) is ultimately unsuccessful.

### 3.3. Gene Expression in Fiskeby III Leaves and Roots

Fiskeby III leaf response to FeD stress at 16D is very different from Mandarin (Ottawa), with only eight DEGs in Fiskeby III leaves compared to the 152 DEGs in Mandarin (Ottawa). Conversely, the number of DEGs in roots is similar between the two genotypes; 37 in Fiskeby III and 22 in Mandarin (Ottawa) (Figure 4). Of the eight DEGs in Fiskeby III leaves, only the bHLH038 homolog, which was discussed earlier, and NAS2 homolog (*Glyma.19G228400*, *At5g56080*), which is involved in moving Fe from roots to shoots, are of obvious importance to FeD or abiotic stress responses. GO analysis of the 37 DEGs in Fiskeby III roots in response to iron stress identified two over-represented terms (GO:0042754, negative regulation of circadian rhythm and GO:0043433, negative regulation of DNA binding TF activity) representing 4 of the 37 DEGs. Given the lack of insights provided by GO analysis, we examined the annotations associated with each of the 37 DEGs. The annotations found that Fiskeby III is responding to FeD conditions by altering the expression of genes known to be involved in abiotic stress responses (eight genes) and known FeD responsive genes (eight genes). All genes known to be involved in FeD responses, except NAS1, are up-regulated in FeD grown plants. The down-regulation of NAS1 in Fiskeby III FeD grown roots mirrors Arabidopsis NAS1 knockouts; which constitutively signal FeD growth conditions and results in accumulating excess Fe in leaf tissues. Thus, down-regulating NAS1 may be increasing Fe uptake and moving existing Fe to leaf tissues for use in photosynthetic processes. This hypothesis is reinforced by the upregulation of genes including *Glyma.12G237367*, which encodes a homolog of FRD3, which transports citrate, in the xylem to transport Fe from roots to shoots [52,86]. In addition, upregulated by FeD is *Glyma.13G168700*, which encodes a formate dehydrogenase. In Arabidopsis, this gene has been hypothesized to regulate not only Fe homeostasis but also biotic and abiotic stress responses [53]. *Glyma.08G169100* is homologous to *At3g12900*, which encodes an enzyme that breaks down scopolitin into fraxetin and a cytochrome P450. In Arabidopsis, Fraxetin is released into the rhizosphere under alkaline conditions where it is able to reduce Fe^3+^ to the usable Fe^2+^ [6,87,88]. This increased iron availability can rescue chlorotic phenotypes, making the up-regulation of *Glyma.08G169100* in Fiskeby III under FeD conditions extremely noteworthy. Examination of previous studies by our lab group found this gene is also up-regulated in Clark roots at 24 hrs, 2days, and 10 days, after FeD, and in Clark roots 24 hrs after P_i_ stress is induced [69,83]. However, expression of *Glyma.08G169100* was not observed in Clark roots or leaves at 30, 60, or 120 min after FeD stress is induced [59], indicating this is a downstream gene, likely turned on in response to calcareous environments. It is noteworthy that this gene is not differentially expressed due to FeD in Mandarin (Ottawa) but is up-regulated in IsoClark after 10 days of FeD stress [69]. While the difference could be attributed to the differences in the timing of the experiments, it is equally possible that the two IDC susceptible genotypes induce different response mechanisms.

### 3.4. Candidate Gene Underlying Gm05 IDC QTL

Given the phenotypes of the VIGS silenced plants under FeS and FeD conditions in both soil and hydroponics, we propose *Glyma.05G001700* is the candidate gene underlying the Gm05 IDC QTL. Mining the results of previous studies in our lab found that in the genotype Clark, *Glyma.05G001700* was differentially expressed in roots in response to iron at both 30 min and 24 h after FeD stress is induced but was no longer differentially expressed after 48 h or 10 days of FeD stress [59,69,83]. Public gene expression data indicates *Glyma.05G001700* is highly up-regulated in roots and nodules but either not expressed or expressed at low levels in all other tissues sampled [29,30]. It is also induced by dehydration and saline stress [89], providing additional evidence that it may play a role in abiotic stress homeostasis. All these results are consistent with the current study where at 16 days of FeD stress, this gene is no longer differentially expressed. However, processes at the onset of FeD stress, including up-regulation of *Glyma.05G001700*, were induced in Fiskeby III to maintain homeostasis and tolerate extended FeD stress conditions.

The closest Arabidopsis homolog of *Glyma.05G001700* is *At1g71140* (AtMATE14). While the annotation of the gene is a DTX MATE transporter, no studies have defined a specific function for the Arabidopsis gene under control conditions, let alone in FeD conditions. Studies in multiple species have found MATE genes play important roles in nutrient deficiency and defense responses [90,91,92]. MATE genes control mechanisms that allow plants to adapt to biotic and abiotic stress conditions, including secreting citrate into the xylem and the rhizosphere, translocating citrate:Fe^2+^ complexes from roots to the shoot, modulating auxin levels to regulate growth, and improving drought stress tolerance by regulating guard cells [91]. The soybean genome encodes roughly 117 MATE transporters that can be organized into four cluster groups [90]. *Glyma.05G001700* (GmMATE27, Liu, et al. 2016) is a member of cluster C2-2, while the best-known MATE gene in Arabidopsis iron deficiency responses, FRD3, is a member of C4-3 [90]. Other members of the C2-2 cluster include AtDTX1, and NtJAT1 [90]. Both AtDTX1 and NtJAT1 are associated with efflux, either antibiotics and toxic compounds (AtDTX1) or nicotine (NtJAT1), suggesting *Glyma.05G001700* is also associated with efflux activity under both FeS) and FeD conditions. *Glyma.05G001700* is minimally expressed in roots and root hairs under normal growth conditions and is known to localize to plasma membranes but not to vacuolar membranes. The expression and localization both support the hypothesis that *Glyma.05G001700* is associated with efflux activity, possibly involved in iron translocation within the root. In soybean, *Glyma.05G001700* has participated in segmental duplication events that includes *Glyma.02G089900* (homologous to AtDTX2) and *Glyma.19G001600* (homologous to AtDTX8) [90]. Importantly, while there are two Fe-effic (IDC) QTL on Gm19 [93,94], neither covers *Glyma.19G001600*. In cotton, over-expression of DTX genes confers tolerance to multiple abiotic stresses, including drought, salt, and cold, likely due to reduced oxidative damage from increased antioxidant enzyme activity and reduced ion leakage [95]. Given the importance of DTX genes in abiotic stress resistance in other species, we hypothesize this gene is the candidate gene underlying the Gm05 IDC QTL. Utilizing RNA-seq on VIGS silenced plants at multiple time points can provide further clues to the role *Glyma.05G001700* plays in the Fiskeby III FeD response.

To better understand the transcriptional regulation of *Glyma.05G001700*, we queried known transcription factor binding sites (TFBS) against the 500bp promoter regions of *Glyma.05G001700* and the homoeologs; *Glyma.02G089900* and *Glyma.19G001600*. Only a single TFBS, for TCX2 (TESMIN/TSO-like CXC 2) was conserved across all three promoter regions. In Arabidopsis, TCX2 (also known as SOL2) regulates the cell cycle and transition from quiescence to proliferation [96]. Regulating the cell cycle in response to stress slows the growth and induces defense and stress tolerance mechanisms to increase plant survival [97]. Historically, modification of the cell cycle has been associated with E2F transcription factor regulation [98,99,100]. It is possible that TCX2 recruits and suppresses E2F TFs, delaying cell cycle progression [101], but other research has shown TCX2 also works to regulate CLAVATA signaling, which regulates stem cell fate [102]. Work by our group has determined that modifying the cell cycle to slow growth is a classic response of Clark (iron efficient) to FeD stress [19,59,69]. Given the prevalence of this system in the plant kingdom, it is highly likely that Fiskeby III leverages a similar approach during periods of FeD stress. Identification of a TCX2 TFBS in the promoter of these three MATE homoeologs offers new experimental avenues to preserve yield in stress conditions.

### 3.5. Comparing Gene Expression in EV and Glyma.05001700 Silenced Plants

Analyzing RNA-seq profiles of VIGS_EV to VIGS_Glyma.05G001700 in FeS conditions and in FeD conditions provides insights into the gene networks affected by silencing *Glyma.05G001700*. In leaves of FeS grown plants, 228 DEGs were identified between VIGS_EV and VIGS_Glyma.05G001700, but only 69 DEGs were identified in FeD grown plants. Because both sets of plants are infected with the VIGS construct, the difference in gene expression patterns is due to the silencing of *Glyma.05G001700*. There were four genes common to the 228 DEGs identified in FeS and 69 DEGs identified in FeD (Figure S1C). In Arabidopsis and other species, two of these (*Glyma.10g192900*, a glutathione S-transferase and *Glyma.15g062500*, a pathogenesis-related protein) are known to interact (Figures S2 and S3). In pepper, the over-expression of a pathogenesis protein increased the expression of glutathione S-transferases, resulting in altered redox balance and increased tolerance to biotic and abiotic stresses [103]. In our data, both these genes are up-regulated in VIGS_Glyma.05G001700 compared to VIGS_EV under both FeS and FeD conditions. These conserved expression patterns, independent of the iron status of the plant, indicate these genes are directly associated with *Glyma.05G001700*. This is also a unique homeostatic process not previously identified in soybean in response to FeD stress, which makes it an exciting avenue for future research.

The identification of so many DEGs in FeS grown plants indicates a role for *Glyma.05G001700* in Fiskeby III iron homeostasis, even in optimal growing conditions. Among the DEGs are two homologs (*Glyma.09G115100* and *Glyma.07G093700*) of *At3g18290*, commonly known as BRUTUS, and two homologs of NRAMP3 (*Glyma.05G101700* and *Glyma.17G165200*), all four of which are down-regulated in VIGS_Glyma.05G001700 compared to VIGS_EV in FeS conditions. The down-regulation of both *BRUTUS* and *NRAMP3* by *Glyma.05G001700* silencing is in direct contrast to findings from Arabidopsis, where down-regulation of BRUTUS increases NRAMP3 expression, which increases iron export from the vacuoles, thus improving tolerance to FeD conditions [104]. Conversely, a suite of genes involved in P_i_ homeostasis, four ferritin homologs (*Glyma.01G124500, Glyma.03G050100, Glyma.07G155200*, and *Glyma.18G205800*), two heavy metal transport proteins *(Glyma.017G065800* and *Glyma.16G032400*), and an NRAMP6 homolog (*Glyma.15G003500*) are all up-regulated in VIGS_Glyma.05G001700 under FeS conditions. Previous work by our group and others has demonstrated macronutrient and micronutrient cross-talk [83,105,106,107,108]. This likely explains how altering Fe availability induces changes in P_i_ homeostasis, transport, and storage [109,110]. The results from this study indicate that by silencing *Glyma.05G001700*, the soybean iron response, P_i_ homeostasis, transport, and storage processes are induced. It is possible that, as a MATE protein, Glyma.05G001700 plays a key role in transporting either iron or iron-associated metabolites and, when silenced, P_i_ transporters are activated in an effort to maintain homeostasis. Alternatively, the up-regulation of P_i_ homeostasis genes might indicate that VIGS_Glyma.05G001700 plants ‘mis-identify’ the nutrient deficiency stress, perhaps because no iron or iron associated metabolites are being transported into or within the plant.

In Arabidopsis, bHLH038 (*At3g56970*) is one of the key regulators of Fe homeostasis [57]. In soybean, the two homologs (*Glyma.03G130400* and *Glyma.03G130600*) are encoded within the Gm03 Fe efficiency QTL. Under FeD conditions, the expression of *Glyma.03G130600*, is up-regulated in VIGS_EV, compared to VIGS_Glyma.05G001700. Interestingly the homolog, *Glyma.03G130400*, is upregulated in Fiskeby III in FeD conditions. These expression patterns indicate that up-regulating *bHLH038* in response to FeD conditions is likely a ‘typical’ FeD response by Fiskeby III. However, this response is eliminated in leaves of VIGS_Glyma.05G001700 under FeD conditions, indicating this gene is affected by the VIGS. Instead, non-canonical genes associated with Fe uptake, transport, and scavenging are up-regulated in VIGS_Glyma.05G001700 compared to VIGS_EV under FeD conditions. These non-canonical genes include *Glyma.12g063600*, an XB3 ortholog that is induced by FeD in Arabidopsis, potentially serving as an iron sensor that indirectly regulates IRT1 [111]. In addition, iron response transporter 3 (IRT3), which normally transports Zn^2+^ ions, but when over-expressed transports Fe^2+^ ions [56] is induced. An NRAMP3 homolog, which is involved with transporting iron from vacuoles to the plastid [112], is also induced in VIGS_Glyma.05G001700 leaves under FeD conditions compared to VIGS_EV. Again, none of these are canonical genes traditionally associated with the soybean iron deficiency response, and none are up-regulated in VIGS_EV plants. It seems that by silencing *Glyma.05G001700*, a ‘backup’ iron response system is induced, again illustrating the resiliency of the soybean genome.

### 3.6. Effect of Iron Treatment on Transcriptome of VIGS Infected Plants

Analyzing gene expression patterns of VIGS_EV in FeS and FeD and VIGS_Glyma.05G001700 in FeS and FeD provides insight into how Fiskeby III VIGS infected plants respond to FeD stress and how silencing *Glyma.05G001700* alters the FeD stress response. Fiskeby III was infected with VIGS_EV to determine the effect of bean pod mottle virus (BPMV) infection has on gene expression patterns in FeS and FeD grown plants. Only 18 DEGs were identified in leaves of VIGS_EV due to FeD stress, and no DEGs were identified in roots (Figure 4). Among the 18 DEGs in leaves are both AtbHLH038 homologs (*Glyma.03G130400* and *Glyma.03G130600*), both of which are up-regulated in FeD conditions. Another eight genes associated with either metal transport or abiotic stress responses are also differentially expressed, accounting for over half of the 18 DEGs. The remaining DEGs are associated with cell wall biosynthesis (3 genes) or have no known function. These results clearly demonstrate that while only *Glyma.03G130400* was differentially expressed in both Fiskeby III leaves, and Fiskeby III VIGS_EV leaves due to iron deficiency. The BPMV infection did not affect the ability of Fiskeby III to respond to FeD stress. In contrast, we expect silencing *Glyma.05G001700* using VIGS would either modify or eliminate the iron deficiency response of Fiskeby III. RNA-seq analysis identified 15 DEGs in VIGS_Glyma.05G001700 leaves due to FeD stress but no DEGs in roots (Figure 4). None of the 15 DEGs from leaves are obviously associated with known Fe uptake or homeostasis pathways. However, five of the genes play important roles in plants exposed to phosphate deficient (-P_i_) growth conditions. Interestingly, all five are down-regulated in FeD grown plants. Previous work by our lab and others has noted the overlap in DEGs responding to FeD and -P_i_ stress [83,105,106,107]. A recent study in Arabidopsis found FeD and -P_i_ stresses induce overlapping but mostly opposing transcriptional responses, highlighting the interactions between FeD and -P_i_ signaling [108]. It is remarkable that silencing Glyma.05G001700 in Fiskeby III eliminates the robust FeD response observed in VIGS_EV plants and down-regulates expression of -P_i_ uptake and homeostasis networks. These results provide clear evidence that *Glyma.05G001700* is an excellent candidate gene underlying the Gm05 IDC QTL.

### 3.7. Conclusions

While the precise role *Glyma.05G001700* plays in conferring tolerance to FeD stress remains unknown, our analyses confirm its importance in the Fiskeby III iron stress response. Further, our analyses suggest clear linkages between iron and phosphate stress responses. It is noteworthy that these responses are only up-regulated under FeS conditions. This suggests that when responses governed by *Glyma.05G001700* expression cannot be utilized due to silenced expression, -P_i_ stress and homeostatic responses are employed instead. The induction of these pathways highlights the unique resilience and flexibility of the Fiskeby III genome to respond to abiotic stresses. They further reinforce the need for additional studies in the Fiskeby III germplasm to understand these responses, thus, they can be leveraged for crop improvement. These results provide novel breeding targets for improved tolerance to various abiotic stresses.

## 4. Materials and Methods

### 4.1. Virus-Induced Gene Silencing (VIGS) Constructs

To develop VIGS constructs for genes within the identified QTL region, we relied on the homologous region of Williams 82, using the Gmax.a4.v1 genome build. Constructs were developed for each of the 10 transcriptionally active genes within the Gm05 QTL. All Constructs were developed using the protocol described in Whitham et al. [113] with the BPMV IA-1033 vector. This version of the VIGS vector was intentionally designed to exhibit viral symptoms to eliminate the need for enzyme-linked immunosorbent assay (ELISA) testing [114]. Primers for *Glyma.05G001700* were developed to amplify a 236bp region of the fifth exon. Primer sequences were F) GAACTGGGGGCAGG and R) CCCCTCTCGCAATCC with XHOI and BAMHI restriction sites added to the F and R primers, respectively. Primers used to develop constructs to test each of the remaining 9 genes within the Gm05 QTL are provided in Table S10. For each of the constructs, sequences were amplified from Williams82 DNA that had been denatured at 94 °C for two minutes followed by 35 PCR cycles (30 s each of 94 °C, 58 °C, 72 °C) followed by a 5 min extension at 72 °C. A 10 μL aliquot of the PCR was used to confirm the appropriate amplicon size. The remainder of the PCR product was cleaned using the Qiagen QIAquick PCR purification kit (Qiagen^®^, Germantown, MD, USA). The PCR product was then digested using 2 μL each of XhoI and BamHI (Promega^®^, Madison, WI, USA) at 37 °C for 2 h, at which point another 2 μL of each restriction enzyme was added for an additional 2 h. After 4 h, the restriction enzymes were inactivated by heating to 65 °C for 15 min. The digested ends were removed from the PCR product using the Qiagen QIAquick PCR purification kit (Qiagen^®^, Germantown, MD, USA). The BPMV IA-1033 vector was digested using the same procedure as the PCR products with the addition of a calf intestinal alkaline phosphatase (CIAP) treatment to prevent self-ligation and subsequent size selection via gel electrophoresis and gel extraction. Digested PCR products and vectors were ligated, and the sequence and orientation were confirmed by sequencing. To generate inoculum for VIGS experiments, BPMV RNA1 (pBPMV-IA-R1M) and the BPMV_Glyma.05G001700 plasmids were co-inoculated via particle bombardment onto Williams 82 unifoliate leaves, 11 days after sowing as previously described [113]. BPMV infection was confirmed 21 days post-bombardment via ELISA (Agdia^®^, Elkhart, IN, USA) PathoScreen BPMV kit for ELISA, PSA 46400/0480). Symptomatic BPMV-infected tissue was collected 4 weeks post-bombardment, lyophilized, and stored at −20 °C. Inoculum was prepared by adding 25mg of lyophilized tissue to 500 μL of 50mM potassium phosphate buffer (pH 7.0). The tissue was disrupted using the TissueLyserII (Qiagen^®^, Germantown, MD, USA) to release the virus. To inoculate experimental plants, unifoliate leaves were dusted with carborundum, 20 μL of the inoculum was applied, and leaves were rubbed, changing gloves between constructs.

### 4.2. Phenotypic Analyses

VIGS constructs were tested in Williams82 (the sequenced genome) and Clark genotypes. For these experiments, 8 inch pots were filled with Metro-Mix 900 potting soil (Sun Grow Horticulture, Agawam, MA, USA). When plants reached the unifoliate stage, plants were rub inoculated as described above with 4 plants per pot. Plants were maintained in a growth chamber with a 16-h photoperiod at 20 °C during the day and 16 °C at night. Plants were watered daily until saturation and fertilized weekly. At 4 weeks post-inoculation (V3) phenotypes, including SPAD, plant height, and shoot weight, were measured. SPAD readings were taken in triplicate across the central leaflet of the V3 trifoliate using a SPAD 502 chlorophyll meter (Spectrum Technologies, Inc., Plainfield, IL, USA). This was repeated twice for each genotype. For the Clark and Fiskeby III FeS and FeD in hydroponics, plants were grown and inoculated as described below but maintained for 21 days. In addition to the phenotypic measurements taken for soil-grown plants, root length, and weight measurements were also taken for hydroponically grown plants.

### 4.3. Hydroponic Growth Conditions

Seeds from Fiskeby III (PI 438471) and Mandarin (Ottawa) (PI 189888) were provided by the University of Minnesota to ensure RNA-seq and VIGS directly mirrored the earlier [15] QTL study. Seeds were surface-sterilized using a 10% sodium hydroxide solution for 3 min, followed by rinsing with distilled deionized water in triplicate. Sterilized seeds were placed on sterile germination paper for 7 days, at which time seedlings were transplanted into hydroponics. The hydroponics was set up exactly as previously described [115,116] with half the plants in iron sufficient (FeS, 100 μM Fe(NO_3_)_3_) and half the plants in iron-deficient (FeD, 50 μM Fe(NO_3_)_3_). After 2 days in hydroponics, seedlings were mature enough for VIGS inoculation; 1/4 of Fiskeby III plants in both FeD and FeS hydroponics were inoculated with VIGS_Glyma.05G001700 construct and ¼ plants inoculated with VIGS_EV construct. The remaining half of the plants were not rub inoculated, to provide samples of Fiskeby III and Mandarin (Ottawa) gene expression responses in FeS and FeD hydroponic conditions. At the time of VIGS inoculation, cotyledons were removed from all plants to force the utilization of iron provided in hydroponics. Plants were maintained in hydroponics for 14 days post-VIGS inoculation (16 days of FeS or FeD hydroponics) till plants were at the V4 stage. Non-destructive phenotyping (SPAD and height measurements) was performed immediately prior to plant harvest. Tissue was collected from all plants (V4 trifoliate and entire root system) and immediately flash-frozen in liquid nitrogen for RNA extraction.

### 4.4. RNA Extraction and Analyses

RNA was extracted from flash-frozen tissue using the Qiagen^®^ RNeasy^®^ Plant Mini Kit (Qiagen^®^, Germantown, MD, USA) according to the manufacturer’s instructions. Contaminating DNA was removed using the Ambion^®^ TURBO DNA-free kit (Ambion^®^, Austin, TX, USA). RNA was further purified and concentrated using the Qiagen^®^ RNeasy^®^ MinElute Cleanup Kit (Qiagen^®^, Germantown, MD, USA). Sample purity and quantity were measured using a nanodrop ND-1000 spectrophotometer (ThermoFisher Scientific, Waltham, MA, USA). RNA was considered to be of good quality if A260/A280 > 1.8. RNA from three biological replicates was submitted to the Iowa State University DNA Facility for sequencing. All reads have been submitted to the NCBI SRA database under BioProject accession PRJNA760474.

RNA-seq libraries were generated from 3ug of total RNA. Subsequent 100bp single-end sequencing was performed using the Illumina HiSeq2500 (Illumina, San Diego, CA, USA). Reads with quality scores over 20 and longer than 30 bases as determined by FastQC [117] were mapped to the soybean genome sequence (Glyma.Wm82.a4.v1 (Glyma 4.0)) using Tophat2 (version 2.1.1) [118] with default parameters except for 10,000 base pair intron maximum length. Uniquely mapped reads were retained using samtools (version 1.3.1) [119]. Data were imported into R-studio (version 0.98.945) for further analysis [120]. The gene feature file (gff) of the soybean genome Glyma.Wm82.a4.v1 (Glyma 4.0) was imported to R using rtracklayer [121], and the number of reads aligning to each gene for each sample was determined using GenomicAlignments [122]. Genes with counts per million < 1 in more than 2 replicates were eliminated from further analysis. Data were normalized using the Trimmed Mean of M (TMM) values [123] in the Bioconductor package edgeR [124]. Specifically, edgeR was used to calculate normalization factors, estimate tagwise dispersion, and determine differential gene expression. Visualizations between replicates were performed using ggplot2 (version3.3.2) [125] to confirm similar gene expression profiles between replicate samples. To identify differentially expressed genes in edgeR, we used a model to account for iron treatment, genotype, and treatment x genotype interaction. For genotype, we considered Mandarin or Fiskeby III when comparing uninfected samples and VIGS_EV or VIGS_Glyma.05G001700 when comparing infected samples. Our model grouped samples by type model.matrix(~0 + Group), and we used contrast statements for comparisons. In all comparisons, a gene was considered differentially expressed if the false discovery rate (FDR) was <0.01. All non-VIGS Fiskeby III and Mandarin (Ottawa) samples (FeS and FeD) were normalized together while all VIGS infected samples (FeS and FeD) were normalized separately. In both cases, leaf and root samples were normalized independently. Since VIGS relies on viral replication, any soybean sequence spliced into the viral vector would be present in extremely high quantities. We used BLASTN to determine whether the spliced sequence would silence any additional MATE genes in the soybean genome; only *Glyma.05G001700* and *Glyma.19G001600* exceeded the 85% identity threshold required for effective gene silencing. Utilizing this information, we examined the expression of *Glyma.05G001700* in VIGS_Glyma.05G001700 silenced plants. This analysis found only 1 of 3 silenced plants in FeD and 2 of 3 silenced plants in FeS with extremely high *Glyma.05G001700* expression. Uninfected samples were removed from downstream analyses. To account for the artificial inflation of *Glyma.05G001700* due to viral replication reads assigned to this gene and its homeolog (*Glyma.19G001600*) were removed from the gene feature file (gff) used for analyses of VIGS plants. All VIGS-infected samples were re-normalized using the modified gff file with *Glyma.05G001700*, and *Glyma.19G001600* removed.

DEGs were assigned annotations using custom perl scripts. The primary Gmax v4 proteins were compared to all available proteins in the *Arabidopsis thaliana* genome (www.TAIR.org, v10) using BLASTP (E > 10^−6^). The best hit and first informative hit were reported. In addition, reported were Gene Ontology (GO) terms associated with each of the best Arabidopsis hits. Over-represented GO terms were identified using custom perl scripts using GO terms assigned as described and uses a Fisher’s exact test [126] with a Bonferroni correction [127] as described in [17]. Over-represented GO terms were used to identify important biological functions and gene classifications. Transcription factors (TFs) were identified using the SoyDB transcription factor database published by [128]. Genotype comparisons were performed using the soybean Genotype Comparison Visualization Tool (GCViT) at SoyBase (https://www.soybase.org/gcvit/, accessed on 19 May 2021). Known interactions between Arabidopsis homologs were identified using the STRING database available at www.string-db.org (accessed on 19 May 2021) [34]. The annotations of the Arabidopsis homologs were examined to determine their biological function. Interactions identified from STRING were visualized in Cytoscape [129], where colors were assigned based on biological function. This visualization facilitated the identification of major biological processes affected by iron deficient growth conditions in different genotypes and by *Glyma.05G001700* silencing. To analyze the promoter region of the 3 homeologous MATE genes, we generated 500 bp promoter regions using custom perl scripts. The program MAST [130,131] with default settings was used with the JASPAR core plant transcription factor binding sites (TFBS) [132] as the binding site query sequences. Only TFBS present in all 3 homeologs was retained, resulting in a single TCX2 TFBS.

## Figures and Tables

**Table 1:** Overrepresented biological process Gene Ontology (GO) terms identified in the 228 DEGs identified between VIGS_EV and VIGS_Glyma.05G001700 FeS leaf samples. Corrected *p*-value was determined after a Fisher’s Exact test followed by a Bonferroni correction to account for repeated sampling.

GO ID	# of DEGs	Corrected *p*-Value	Description
GO:0016036	16	3.02 × 10^−6^	Cellular response to phosphate starvation
GO:0030643	3	0.005	Cellular phosphate ion homeostasis
GO:0019375	10	0.005	Galactolipid biosynthetic process
GO:0055072	6	0.007	Iron ion homeostasis
GO:0006879	4	0.011	Cellular iron ion homeostasis
GO:0006091	7	0.012	Generation of precursor metabolites and energy
GO:0006817	5	0.018	Phosphate ion transport
GO:0015979	13	0.020	Photosynthesis
GO:0010043	7	0.035	Response to zinc ion

**Table 2:** Overrepresented biological process Gene Ontology (GO) terms identified in VIGS_Glyma.05G001700 leaf samples in response to iron availability (FeS vs. FeD). Corrected *p*-value was determined after a Fisher’s Exact test followed by a Bonferroni correction to account for repeated sampling.

GO ID	# of DEGs	Corrected *p*-Value	Description
GO:0019375	6	0.0001	Galactolipid biosynthetic process
GO:0016036	6	0.001	Cellular response to -P_i_ stress
GO:0030643	2	0.002	Cellular phosphate ion homeostasis

## Data Availability

In Datasets associated with this study can be found in the short-read archive (SRA) database (http://www.ncbi.nih.gov/sra) under BioProject accession PRJNA760474.

## Supplementary Materials

The following are available online at https://www.mdpi.com/article/10.3390/ijms222011032/s1.
