# Supplementary figures and images for: Mining Fiskeby III and Mandarin (Ottawa) Expression Profiles to Understand Iron Stress Tolerant Responses in Soybean
Source: Int J Mol Sci. 2021 Oct 13;22(20):11032. doi: 10.3390/ijms222011032 (PMC8537376; doi:10.3390/ijms222011032)

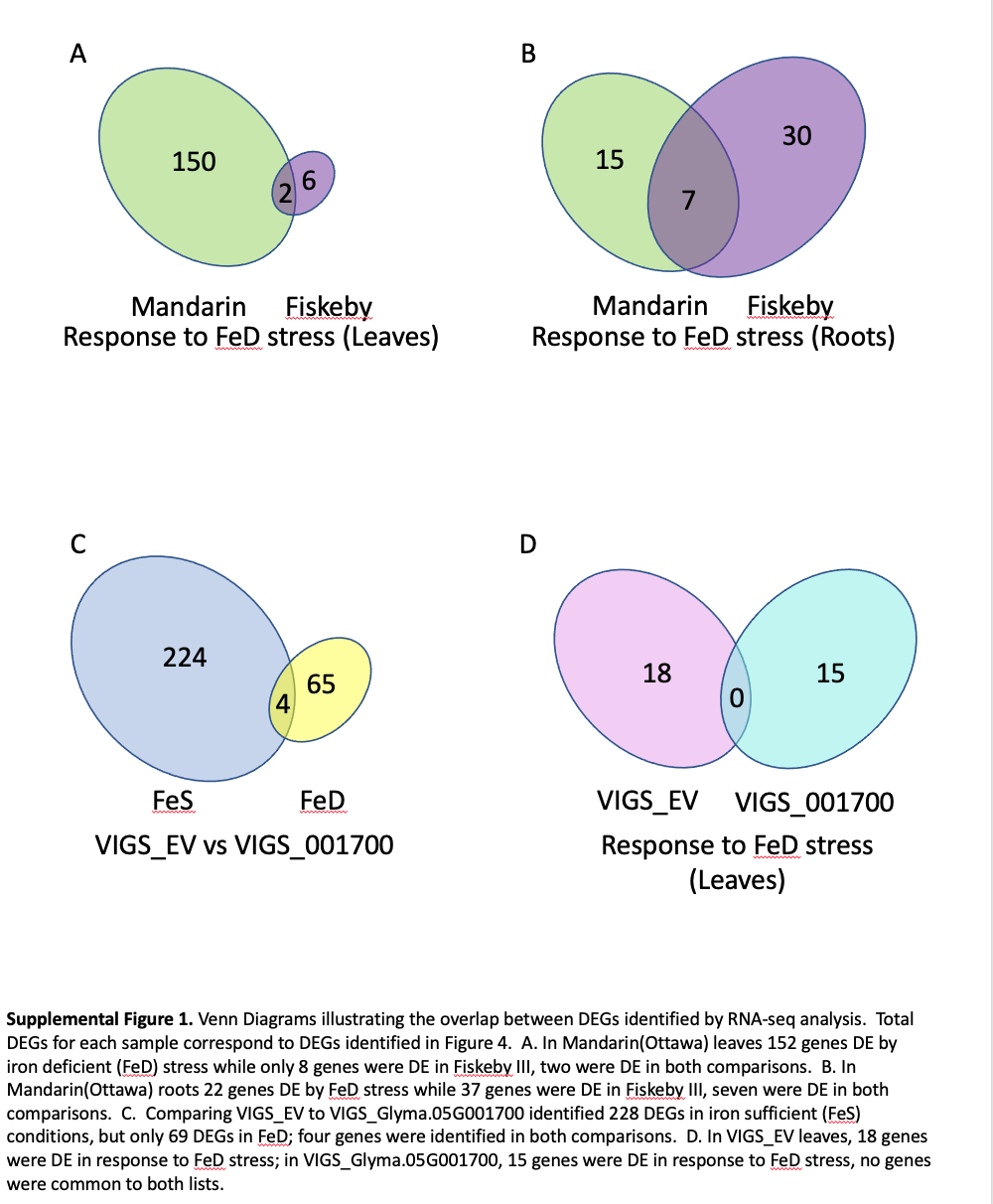

Supplement: Supplementary file 1 [file ijms-22-11032-s001.zip › Figure S1.png]

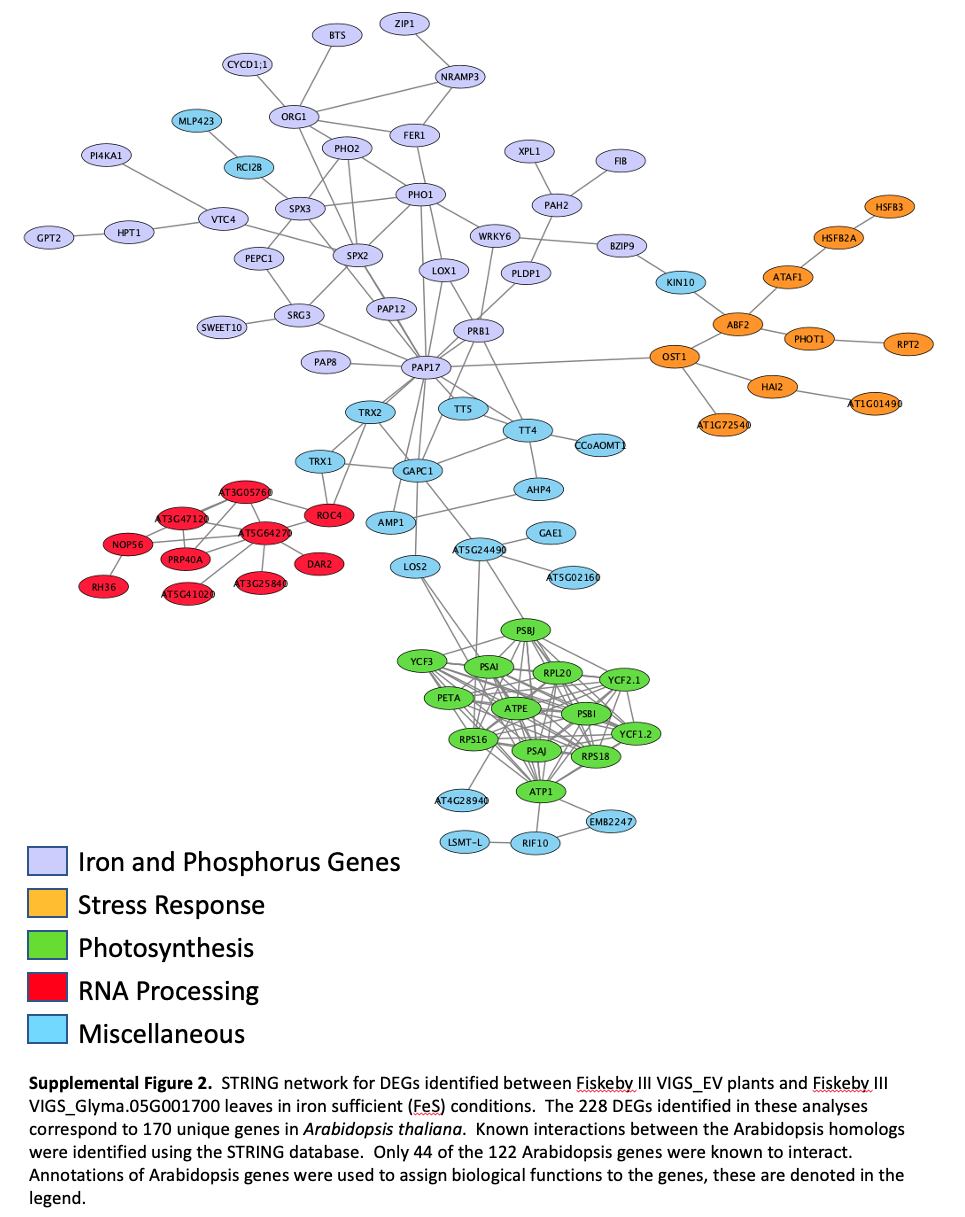

Supplement: Supplementary file 1 [file ijms-22-11032-s001.zip › Figure S2.png]

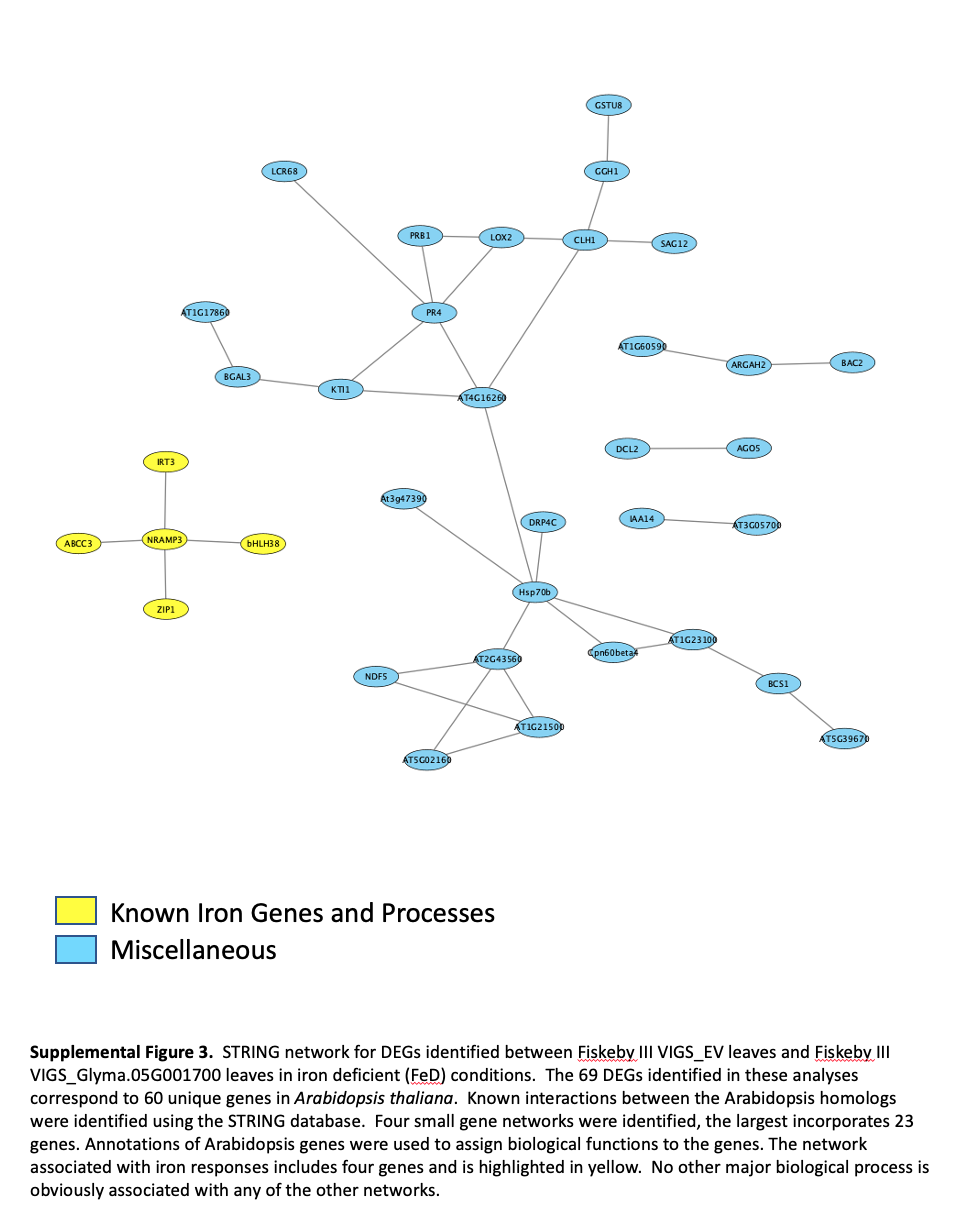

Supplement: Supplementary file 1 [file ijms-22-11032-s001.zip › Figure S3.png]
